# MicroRNA-21 and Risk of Severe Acute Kidney Injury and Poor Outcomes after Adult Cardiac Surgery

**DOI:** 10.1371/journal.pone.0063390

**Published:** 2013-05-23

**Authors:** Juan Du, Xiaoqing Cao, Liang Zou, Yi Chen, Jin Guo, Zujun Chen, Shengshou Hu, Zhe Zheng

**Affiliations:** 1 State Key Laboratory of Cardiovascular Medicine, Fuwai Hospital, National Center for Cardiovascular Disease, Chinese Academy of Medical Sciences, Peking Union Medical College, Beijing, China; 2 Department of Surgery, Fuwai Hospital & Cardiovascular Institute, Chinese Academy of Medical Sciences, Peking Union Medical College, Beijing, China; 3 Key Laboratory of Cardiac Regenerative Medicine, Ministry of Health, National Center for Cardiovascular Diseases, Beijing, China; CNR, Italy

## Abstract

**Background:**

Severe acute kidney injury (AKI) after cardiac surgery is associated with poor clinical outcomes. This study evaluated the potential use of miR-21 as a risk marker for postoperative AKI progression and other poor outcomes.

**Methodology/Principal Findings:**

The study included 120 adult patients undergoing cardiac surgery: 40 non-AKI controls, 39 patients with progressive AKI, and 41 with non-progressive AKI. Urine and plasma levels of miR-21 were assessed by quantitative real-time PCR (RT-qPCR). Associations between miR-21 levels and AKI progression were determined by estimating areas under receiver operating characteristic curves (AUC). We demonstrated that up-regulated urine and plasma levels of miR-21 in patients with AKI were both associated with AKI progression. The AUCs for urine and plasma levels of miR-21 associated with established AKI were 0.68 (95%CI: 0.59–0.78) and 0.80 (95%CI: 0.73–0.88), respectively. Multiple logistic regression analysis, adjusting for clinical variables, indicated that the prognostic predictive power of urine and plasma miR-21 levels for AKI progression were represented by AUCs of 0.81 (95%CI: 0.72–0.91) and 0.83 (95%CI: 0.74–0.92), respectively. Urinary and plasma miR-21 levels also predicted the need for postoperative renal replacement therapy (RRT), development of Acute Kidney Injury Network (AKIN) stage 3 AKI, 30-day in-hospital mortality and prolonged stay in hospital or ICU. Urine miR-21 was a better outcome predictor than plasma miR-21, being associated with higher (1.4- to 2.6-fold) unadjusted odds ratio for progression of AKI and other poor outcomes.

**Conclusions:**

Urinary and plasma miR-21 are associated with severe AKI and other poor postoperative outcomes of cardiac surgery, indicating their potential use as prognostic markers.

## Introduction

Acute kidney injury (AKI) is a common and potentially serious postoperative complication of cardiac surgery [1], [2]. The incidence of cardiac surgery-associated AKI (CSA-AKI) varies from 5% to 45% depending on the diagnostic system, the type of cardiac surgery and the mode of detection [3]–[5]. Majority of patients with CSA-AKI do not experience severe AKI. Indeed, a large cohort study (TRIBE-AKI) found that 86.5% of patients with AKI remained in the mild stage of the disease during their postoperative hospital stay [6]. According to the Kidney Disease Improving Global Outcomes (KDIGO) criteria, these low-risk patients do not require therapeutic intervention [7]. With increased severity of AKI, the risks of chronic kidney disease (CKD), end-stage renal disease (ESRD) and death increase accordingly [8]–[10]. It has been estimated that the risk of mortality is increased 2- to 5-fold in patients whose plasma creatinine increases more than 2-fold from baseline [11]. Indentifying high-risk patients with kidney function loss, prior to serum creatinine levels demonstrating significant changes is an important clinical objective.

However, most studies focus on early detection of AKI other than prediction of progression of AKI. Several clinical scores have been used to predict severe AKI after cardiac surgery [12]. Regrettably, their predictive power is limited as they are based on preoperative factors only. Furthermore, new biomarkers proposed for early detection for AKI, are of limited value in evaluating the degree of kidney injury and predicting of prognosis, and at best exhibit moderate predictive capacity with AUCs that are consistently <0.8 [6], [13].

MicroRNAs (miRNAs) are endogenous, non-coding and small (18–22 nucleotides) RNA molecules. They are tissue-specific, easily amplified by signal pathways, and have wide-ranging (patho)physiological effects. They are remarkably stable in blood and other body fluids and have emerged as novel biomarkers that reflect various disease states [14]–[17]. MiRNAs have recently been identified that play a role in renal cancer, diabetic nephropathy and hypertensive renal injury [18]–[20]. In clinical studies, in intensive care unit (ICU) setting, plasma miR-210 level has been revealed to be up-regulated in AKI patients upon the time point of the first renal replacement therapy (RRT) initiation [21]. However, as 62% patients in the study were anuric/oliguric at the time of sample collection, miR-210 might not be an ideal biomarker for predictive purposes. Therefore, there is a need to find other miRNAs that are involved in the etiology of CSA-AKI and have earlier response to kidney injury. In Vivo animal studies, miR-21 expression was found to increase in different models of acute renal injury. Especially in ischemia-reperfusion injury (IRI) models, up-regulation of miR-21 occurred in an early time-point and lasted for several days [22]–[25]. Indeed, IRI has been identified as one of the most important injury pathways involved in CSA-AKI [26].

In the present study, we hypothesized that the levels of circulating miR-21 could be used to detect and monitor the pathological development of acute kidney injuries after cardiac surgery. Here we reported that the blood and urine levels of miR-21 might be associated with risk for AKI progression in patients undergoing cardiac surgery.

## Materials and Methods

### Ethics Statement

The study has been approved by the Ethical Committee of Fu Wai Hospital, and adhered to the tenets of the Declaration of Helsinki. In addition, all patients provided informed written consent prior to cardiac surgery was carried out.

### Study Design and Patients

The study population included adult patients, referred for cardiac surgery at the ninth ward of Fu Wai Hospital between November 2011 and June 2012. All patients had at least one preoperative risk factor for AKI according to the Cleveland score [27]. Preoperative creatinine values were obtained within the 2 weeks prior to surgery. If measured more than once the highest value was used as a baseline. The patients were divided into two groups according to whether or not developing AKI after cardiac surgery. The AKI group (n = 80) had Stage 1 or 2 AKI defined by Acute Kidney Injury Network (AKIN) criteria with a postoperative increase in plasma creatinine ≥50% baseline or ≥0.3 mg/dL [28]. Patients showing no evidence of unfavorable postoperative increase in plasma creatinine formed the non-AKI group (*n* = 40). Exclusion criteria included end-stage renal disease, cancer and infection diseases. Patients whose initial AKI diagnosis was stage 3 (the highest stage of AKI) and those with aggravated preoperative AKI were not included in the AKI group in order to avoid confounding results for outcome evaluation. Those Patients who developed AKI after postoperative sample collection were excluded from the non-AKI group.

### Sample Collection and MicroRNA Extraction

Baseline urine and blood samples were collected at the time of enrollment. Follow up samples were collected as soon as possible from patients in the AKI group and on the morning of the first postoperative day from patients in the non AKI group. All samples were processed within 1 h after collection. Urine samples were centrifuged at 4°C at 10000 g for 10 min and plasma samples were centrifuged at 3000 rpm for 15 min. Aliquots of the samples were added to RNase/DNase-free tubes and stored at −80°C. Total RNA was extracted from plasma and urine samples, using the miRVana PARIS Kit (AM1556; Applied Biosystems, CA, USA) according to the manufacturer's instructions. Briefly, 400 µL of urine or EDTA plasma sample was added to an equal volume of denaturing solution and incubated on ice for 5 min. The plasma and urine samples were spiked with 10 ng of foreign *Caenorhabditis elegans* miR-39 (cel-miR-39, Applied Biosystems), which lacked sequence homology to human miRNAs. Acid-phenol chloroform solution (800 µL) was added to the denatured samples to create an aqueous RNA phase, which was removed and passed through glass-fiber filters to bind the RNA. The resulting RNAs were eluted in 50 µL of nuclease-free water.

### Reverse Transcription (RT) and Quantitative Real-time PCR (qPCR)

4.5 µl RNA sample was reverse transcribed with TaqMan miRNA reverse trascription Kit (Applied Biosystems) using prespecified miRNA sequences for miR-21 and cel-39 (ABI), according to the manufacturer's instructions. The reaction volume was set to 10 µl. After reverse transcription, miR-21 and cel-miR-39 expression in 2 µL samples was detected by quantitative polymerase chain reaction (PCR) using Taqman miRNA assay kits (ABI). For each qPCR reaction, the total reaction volume is 20 µl. After 10 minutes incubation at 95°C, amplification was performed for 40 cycles at 95°C for 15 seconds and at 60°C for 1 minute. Each sample was run in triplicate in each qPCR reaction. The 2^−Δct^ method was used to evaluate the relative expression of miRNA [29]. Threshold cycle (Ct) values of miR-21 were normalized to cel-miR-39 and expressed as Δct, defined as the ratio of the miRNA of interest relative to internal control gene. Levels of miR-21 were calculated as 2^−Δct^ (i.e.: 2^− (CT[miR−21] −CT[cel−miR−39])^). Group comparisons were based on mean 2^−Δct^ ± standard deviations (SD).

### Study Endpoints

Baseline characteristics, surgical details, and postoperative factors were recorded in accordance with procedures of the Society of Thoracic Surgeons data collection tool. Estimated glomerular filtration rate (eGFR) was calculated using the Chinese Modification of Diet in Renal Disease (MDRD) equation [30]. Dipstick urinalysis, was used to classify proteinuria as being mild (trace or 1+), or heavy (≥2+) proteinuria.

The primary outcome was the progression of postoperative AKI, defined as development to a higher AKIN stage (from stage 1 to either stage 2 or 3 or from stage 2 to 3) after the time of the postoperative sample collection. Secondary outcomes were the need for renal replacement therapy (RRT), 30 day in-hospital mortality, and length of stay in hospital and ICU after surgery.

### Statistical Analysis

Statistical analysis was performed using SAS version 9.2 (SAS Institute, Inc, Cary, NC). Continuous variables were expressed as means ±SD and between-group differences were analyzed using one-way analysis of variance. Categorical variables were compared using Pearson chi-square or Fisher’s exact tests. Multiple logistic regression analysis was used to establish a clinical risk prediction model. The clinical model included Cleveland Score [27], preoperative eGFR (30–60, 60–90 or >90 ml/min), operation time, and cardiopulmonary bypass time (>120 min).

The performance of biomarkers for outcome prediction was determined using receiver operating characteristic (ROC) curve analysis. Statistically significance was defined for univariate ROC curves when the area under the curves (AUC) differed from 0.5 by z test determination. Odds ratios (OR) with 95% confidence intervals (CI) was tested to determine the associations between biomarkers and selected outcomes. Best cut-offs were selected as the values that minimized the geometric distance from both 100% sensitivity and 100% specificity on the ROC curves [31]. All statistical tests were two-sided and values of P values less than 0.05 were considered to be statistically significant.

## Results

### Patients Characteristics

Among 80 adults who suffered from AKI after cardiac surgery, 59 (73.7%) patients were diagnosed within the first postoperative day, and 21 (26.3%) developed AKI the following day. Eight (10%) patients has an initial diagnosis of AKI Stage 2, 72 (90%) patients had Stage 1 disease at the time of the postoperative sample collection. Sixteen (20%) patient progressed from Stage 1 or 2 to 3 and 23 (29%) progressed from Stage 1 to 2 after sample collection.

Based on above findings these patients were divided into progressors (n = 39) and non-progressors (n = 41). The other forty patients undergoing cardiac surgery without AKI served as a control group. There was no statistically significant difference in sample collecting times between these three groups.

The baseline characteristics and perioperative risk factors that may influence AKI development in the three groups of patients were summarized in Table 1 [32]–[34]. Preoperative and surgical risk factors were based on the Cleveland score [27]. As shown in our data, there was no significant difference in Cleveland scores between non-AKI patients and AKI patients without progression, since the combination of clinical factors is mostly used for predicting the risk of dialysis therapy and doubling of serum creatinine level after surgery, rather than mild AKI. However, the score was significantly higher in the group with AKI progression than in the non-progressive and non-AKI groups. One-way ANOVA indicated that patients with AKI progression were more likely to be female and to have undergone non-elective or complex surgeries than patients in the other two groups. They also had a higher use of postoperative, intra-aortic balloon pumps (IABP) and diuretics, and significantly longer operation and cardiopulmonary bypass times. Although none of these patients with progressed AKI had a baseline eGFR less than 30 mL/min/1.73 m^2^, their baseline renal eGRF was significantly lower than the other two groups.

**Table 1:** Clinical characteristics for patients who did not develop AKI and those with and without AKI progression.

	non AKI	AKI progressed^a^		*P* Value
	n = 40	No (n = 41)	Yes (n = 39)	
**Demographics and preoperative factors**				
age (years)	59.9±10.1	59.2±13.8	61.2±13.3	0.768
female, n (%)	10(25)	10(24)	16(41)	0.187
BMI (kg/m^2^)	25.6±3.7	24.3±3.6	24.3±3.4	0.193
diabetes, n (%)	14(35)	10(24)	12(31)	0.576
hypertension, n (%)	20(50)	27(66)	26(67)	0.228
congestive heart failure, n (%)	4(10)	5(12)	5(13)	0.919
COPD, n (%)	2(5)	3(7)	2(5)	0.882
myocardial infarction, n (%)	20(50)	20(49)	19(49)	0.992
ejection fraction (%)	57.9±8.4	56.2±10.2	55.3±9.3	0.468
preoperative SCr (mg/dl)	84.7±20.9	84.3±18.7	90.4±20.8	0.323
preoperative eGFR^b^ (ml/min/1.73 m^2^)	83.8±23.6	80.9±26.8	69.3±24.7	0.028
30∼60, n (%)	6(14)	9(22)	17(44)	0.011
60∼90, n (%)	21(53)	15(37)	16(41)	0.331
>90, n (%)	13(33)	17(41)	6(15)	0.036
preoperative proteinuria^c^				
mild, n (%)	4(10)	6(15)	8(21)	0.424
heavy, n (%)	2(5)	2(5)	3(8)	0.834
cardiac catheterization in last 72 h, n (%)	5(13)	4(10)	6(15)	0.749
preop IABP, n (%)	1(3)	1(2)	2(5)	0.749
**Surgical factors**				
elective operation, n (%)	35(88)	35(85)	32(82)	0.792
redo cardiac surgery, n (%)	1(3)	3(7)	1(3)	0.461
surgery type				
CABG, n (%)	31(77)	31(76)	22(57)	0.077
valve, n (%)	6(15)	3(7)	6(15)	0.465
CABG and valve, n (%)	2(5)	3(7)	7(18)	0.124
other, n (%)	1(3)	4(10)	4(10)	0.338
operation time (h)	5.7±1.4	5.3±1.3	6.3±2.3	0.026
off CPB surgery, n (%)	5(13)	7(17)	7(18)	0.774
CPB time (min)	124.8±39.8	116.2±41.6	168.3±94.9	0.002
aortic cross-clamp time (min)	86.2±26.9	80.9±34.5	106.5±70.3	0.075
**Cleveland score**	1.83±1.43	1.88±1.62	2.64±1.58	0.035
**Postoperative factors**				
vasoconstrictors administration, n (%)	10(25)	15(37)	13(33)	0.514
inotropes administration, n (%)	20(50)	21(51)	20(51)	0.992
diuretics administration, n (%)	5(13)	6(15)	11(28)	0.148
red blood cells transfusion (units)	3.1±1.4	2.9±1.5	3.5±2.6	0.66
plasma transfusion (ml)	400.0±106.9	433.3±187.5	400.0±227.1	0.891
postop IABP, n (%)	2(5)	2(5)	4(10)	0.55

COPD, chronic obstructive pulmonary disease; SCr, plasma creatinine; eGFR, estimated glomerular filtration rate; IABP, intra-aortic balloon pump; CABG, coronary artery bypass grafting; CPB, cardiopulmonary bypass.

Continuous variables were expressed as means ±SD.

a AKI progression defined by worsening of AKIN stage from original diagnosis of AKI.

b Estimate eGFR by the Chinese MDRD equation.

c Proteinuria by dipstick urinalysis, mild (trace or 1+) and heavy (≥2+).

As shown in Table 2, there was no statistically significant between group difference in mild proteinuria and urine output on the day of AKI diagnosis. Poor clinical outcomes mainly occurred in progressor group. The length of ICU and hospital stay was also higher in patients with AKI progression than in those in the other two groups.

**Table 2:** Outcomes in patients with and without AKI.

	non AKI	AKI progressed^a^		*P* Value
	n = 40	No (n = 41)	Yes (n = 39)	
sample collection time (postop h)	18.5±6.8	19.5±10.4	20.4±7.9	0.616
**Time of sample collection**				
SCr (mg/dl)	1.1±0.3	1.5±0.3	1.6±0.4	<0.0001
percent change in SCr^b^ (%)	9.5±12.5	58.8±23.1	62.9±33.5	<0.0001
urine output (ml/kg per h)	3.0±1.1	2.9±1.1	3.0±1.2	0.99
proteinuria^c^				
mild, n (%)	5(13)	5(12)	5(13)	0.996
heavy, n (%)	0	2(5)	7(18)	0.007
plasma miR-21 [2^−△ct^, (*10^−4^)]	4.03±3.87	9.41±7.71	20.96±18.34	<0.0001
urine miR-21 [2^−△ct^, (*10^−4^)]	1.67±1.51	2.41±1.98	5.06±3.70	<0.0001
**AKI progression**				
maxSCr (mg/dl)	1.2±0.3	1.3±0.3	3.2±1.7	<0.0001
△SCr (mg/dl)	0.2±0.1	0.7±0.2	2.2±1.6	<0.0001
days to maxSCr	1.7±0.8	2.4±0.5	4.3±2.6	<0.0001
duration of AKI (days)	0	3.0±2.5	9.2±7.2	<0.0001
**Other outcomes**				
received RRT, n (%)	0	0	6(15)	0.001
Length of ICU stay (days),	2.4±1.2	3.6±2.0	6.2±3.0	<0.0001
Length of hospital stay (days)	7.0±1.9	9.8±5.7	14.0±8.1	<0.0001
30 day in-hospital mortality, n (%)	0	0	7(18)	<0.0001

SCr, plasma creatinine; △SCr, the difference between the peak postoperative and baseline plasma creatinine levels; maxSCr, maximum plasma creatinine; RRT, renal replacement therapy; ICU, intensive care unit.

a AKI progression defined by worsening of AKIN stage from original diagnosis of AKI.

b Defined as percent change in postoperative plasma creatinine from baseline at the time of AKI diagnosis.

c Proteinuria by dipstick urinalysis, mild (trace or 1+) and heavy (≥2+).

### Analysis of miR-21 in Subjects

**Figure 1:**
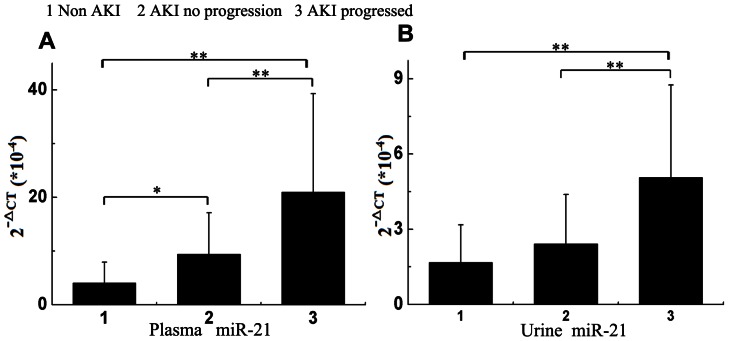
Comparison of plasma and urine microRNA-21 levels in patients with AKI and in non-AKI controls. Expression of miR-21 in EDTA-plasma (A) and urine (B) obtained from patients with progressing AKI (*n* = 39), AKI without progression (*n* = 41) and non-AKI controls (*n* = 40) as determined by TaqMan PCR. Levels of miR-21 were expressed as the 2^−Δct^ value, multiplied by 10^−4^. After post hoc analysis, only the association urinary miR-21 levels between non-AKI and non-progressive AKI groups failed to reach statistical significance (*P* = 0.19). *P<0.05 and **P<0.0001.

The levels of urinary and plasma miR-21 expression increased in parallel with progression of AKI (Figure 1). Except for differences in urinary miR-21 level between non-AKI patients and non- progressors, all other between-group comparisons were statistically significant.

**Figure 2:**
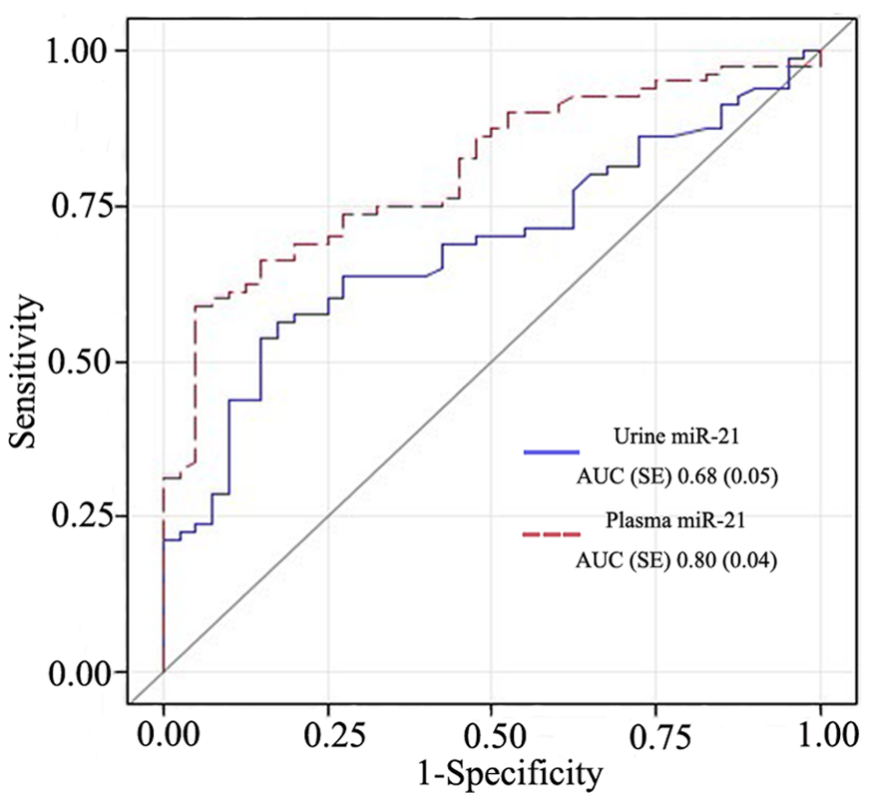
Performance of urine miR-21 and plasma miR-21 for established AKI detection. Receiver-operating characteristic (ROC) curves show the distinguishing performance of urine and plasma miR-21 levels that obtained from 40 patients in the non AKI group and 80 patients in the AKI group.

Our results verified the performance of miR-21 for detecting established AKI. Both plasma and urinary miR-21 levels measured as soon as AKIN criteria for Stage 1 AKI were met, were significantly higher than in the non-AKI patients (p<0.0001). The levels expressed as mean 2^−Δct^ ± SD were 1.67±1.51 versus 3.70±3.22 for urine miR-21 and 4.03±3.87 versus 15.04±15.02 for plasma miR-21 from 40 patients in the non AKI group and 80 patients in the AKI group, respectively. ROC curve analysis revealed that miR-21 can be used to identify AKI (Figure 2). The area under the ROC curves (AUC-ROC) were 0.68 (95% CI: 0.59–0.78) for urine and 0.80 (95%CI: 0.73–0.88) for plasma, indicating that plasma miR-21 was a more reliable marker than urine miR-21 for detection of established AKI.

### MiR-21 as a Predictor of AKI Progression

**Figure 3:**
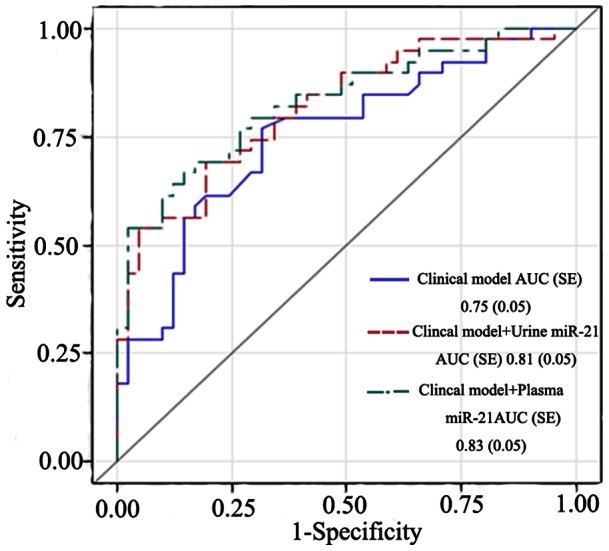
Predictive performance of the urine and plasma value of miR-21 for the progression of AKI. Receiver-operating characteristic (ROC) curves show the predictive performance of the clinical model, clinical model+urine miR-21, and clinical model+plasma miR-21. MiR-21 levels obtained from progressors (*n* = 39) and non-progressors (*n* = 41) of AKI group.

Multiple logistic regression analysis identified urine miR-21, plasma miR-21 and preoperative eGFR as independent prognostic risk factors for the progression of AKI (Table 3). A clinical model including Cleveland Scores, preoperative eGFR, operation time, and CPB time was established to adjust for differences in covariables between groups. The AUC-ROC for clinical model alone was 0.75 (95% CI 0.65–0.86). Urinary and plasma miR-21 provided moderate levels of prediction for AKI prognosis with AUCs of 0.72 (95% CI: 0.61–0.83) and 0.72 (95% CI: 0.60–0.83), respectively. After adjustment using the clinical model, the accuracy increased to 0.81 (95% CI: 0.72–0.91) for urinary miR-21 and 0.83 (95% CI: 0.74–0.92) for plasma miR-21 (Figure 3). The ORs for AKI worsening were 1.4 (95%CI: 1.1–1.7) for urinary miR-21 and 1.1 (95%CI: 1.0–1.2) for plasma miR-21. A non-significant association was demonstrated between AKI progression and marked postoperative proteinuria, or percentage change in serum creatinine on the first day of meeting AKI diagnostic criteria.

**Table 3:** Predictive power for AKI progression (urine and plasma miR-21 versus routine measurements).

Biomarkers	unadjusted				Adjusted a			
	AUC (SE)	95%CI	OR(95%CI)	*P* Value	AUC (SE)	95%CI	OR(95%CI)	*P* Value
Urine miR-21	0.72 (0.06)	0.61–0.83	1.38(1.14. - 1.67)	0.001	0.81 (0.05)	0.72–0.91	1.35(1.10–1.67)	0.005
Plasma miR-21	0.72 (0.06)	0.60–0.83	1.08(1.03–1.14)	0.002	0.83 (0.05)	0.74–0.92	1.10(1.03–1.17)	0.003
SCr	0.62 (0.06)	0.50–0.75	1.02(1.00–1.03)	0.06	0.76 (0.05)	0.66–0.87	1.01(0.99–1.03)	0.48
Percent change in SCr^b^	0.50 (0.07)	0.37–0.63	1.00(0.99–1.02)	0.52	0.79 (0.05)	0.69–0.89	1.02(1.00–1.04)	0.132
Heavy proteinuria^c^	0.57 (0.04)	0.50–0.63	4.27(0.83–21.98)	0.08	0.77 (0.05)	0.66–0.87	3.92(0.65–23.55)	0.135

SCr, plasma creatinine; AUC, area under the curve; CI, confidence interval; OR, odds ratio.

a Adjusted for the clinical mode, which include Cleveland score, preoperative eGFR(30–60,60–90,and >90,), operation time, and CPB time >120minutes (clinical model AUC = 0.75).

b Defined as percent change in postoperative plasma creatinine from baseline at the time of AKI diagnosis.

c Proteinuria by dipstick urinalysis, heavy (≥2+).

### MiR-21 as a Predictor of Poor Outcomes

As shown in Table 4, urinary and plasma miR-21 expression predicted the postoperative need for RRT, as well as 30-day in-hospital mortality, and prolonged stay in hospital and ICU after surgery. The accuracy of predicting postoperative RRT was 0.99 (95%CI: 0.96–1.00, p = 0.003) with urinary miR-21 and 0.97 (95%CI: 0.90–1.00, P = 0.005) with plasma miR-21. Both AUCs for predicting 30 day in-hospital mortality were higher than 0.85. Urinary miR-21 performed better than plasma miR-21 as a predictor for adverse clinical outcomes with unadjusted ORs being 2.6-fold (95%CI: 1.4–4.9), 1.7-fold (95%CI: 1.3–2.3) and 1.6-fold (95%CI: 1.3–2.0) higher than for plasma miR-21 in predicting RRT, in-hospital death, and the development of Stage 3 AKI. Table 5 summarizes the performance characteristics of miR-21 as a predictor of the tested outcomes in patients who had AKI at the time of sample collection.

**Table 4:** Urine and Plasma miR-21 with other poor outcomes.

outcomes	Urine miR-21 (2^−△ct^ value)				Plasma miR-21 (2^−△ct^ value)			
	AUC (SE)	95%CI	OR(95%CI)	*P* Value	AUC (SE)	95%CI	OR(95%CI)	*P* Value
received RRT	0.99 (0.01)	0.96–1.00	2.59(1.37–4.92)	0.003	0.97 (0.03)	0.90–1.00	1.20(1.06–1.36)	0.005
Death a	0.93 (0.04)	0.85–1.00	1.68(1.25–2.27)	<0.001	0.88 (0.08)	0.72–1.00	1.12(1.05–1.20)	<0.001
AKIN stage 3	0.82 (0.07)	0.69–0.95	1.56(1.25–1.95)	<0.001	0.81 (0.06)	0.69–0.93	1.10(1.04–1.15)	<0.001
Prolonged hospital stay b	0.73 (0.06)	0.63–0.84	1.40(1.15–1.70)	<0.001	0.71 (0.06)	0.60–0.82	1.06(1.02–1.11)	0.007
Prolonged ICU stay c	0.72 (0.06)	0.61–0.83	1.28(1.08–1.52)	0.004	0.67 (0.06)	0.55–0.79	1.05(1.01–1.10)	0.015

RRT, renal replacement therapy; AKIN, Acute Kidney Injury Network; ICU, intensive care unit.

a Death defined as 30 day in-hospital mortality.

b Defined as discharge >10 days from sample collection.

c Defined as length of ICU stay >4 days.

**Table 5:** Performance characteristics of urine and plasma miR-21 as prognostic AKI biomarkers among patients with AKI at the time of sample collection (n = 80).

Outcome	Best Cut-off (2^−△ct^ value)	n(%)^a^	Sensitivity(%)	Specificity(%)	PPV(%)	NPV(%)
AKI progression	urine≥3.90	34(42.5)	59.0	73.2	67.7	65.2
	plasma≥10.99	40(50)	66.7	65.9	65.0	67.5
received RRT	urine≥9.04	7(8.8)	100.0	98.6	85.7	100.0
	plasma≥43.65	5(6.3)	83.3	100.0	100.0	98.7
Death b	urine≥5.77	18(22.5)	85.7	83.6	33.3	98.4
	plasma≥19.79	21(26.3)	85.7	79.5	28.6	98.3
AKIN stage 3	urine≥4.89	23(28.8)	68.8	81.3	47.8	91.2
	plasma≥12.38	33(41.3)	81.3	68.8	39.4	93.6
Prolonged hospital stay c	urine≥3.00	40(50)	65.7	62.2	57.5	70.0
	plasma≥7.89	50(62.5)	77.1	48.9	54.0	73.3
Prolonged ICU stay d	urine≥2.73	43(53.8)	71.1	61.9	62.8	70.3
	plasma≥10.49	44(55)	68.4	57.1	59.1	66.7

PPV, positive predictive value; NPV, negative predictive value; RRT, renal replacement therapy; AKIN, Acute Kidney Injury Network; ICU, intensive care unit.

a The percentage and number of patients who were above and equal to the best PPV cut-off.

b Death defined as 30 day in-hospital mortality.

c Defined as discharge >10 days from sample collection.

d Defined as length of ICU stay >4 days.

## Discussion

In this study, we demonstrated the use of plasma and urinary miR-21 as prognostic biomarkers of AKI following cardiac surgery. Our findings indicate that both urine and plasma miR-21 levels were able to identify established AKI after cardiac surgery, and that the detection of miR-21 in urine and plasma is feasible. We also showed that urine and plasma miR-21 at the time of creatinine-based diagnosis of AKI were strong predictors of worsening of AKI and other unfavorable outcomes. The levels of miR-21 in EDTA-plasma were higher than in urine, but urinary miR-21 was a better predictor of progression of AKI and other adverse clinical outcomes than plasma miR-21, for being associated with higher (1.4- to 2.6-fold) unadjusted odds ratio for these selected outcomes.

Our study highlighted that miR-21 levels increased both in plasma and urine with the severity of AKI, although, it was unclear whether miR-21 reflected the severity of kidney injury or whether it served a protective function. As shown in a previous animal study, miR-21 controlled necrosis and apoptosis of renal tubular epithelial cells (TEC) and promoted cellular proliferation in response to rat renal IRI [22]. It is thus conceivable that this small molecule could be released from kidney and play a protective role in AKI. Similarly, other studies have revealed that miR-21 controls human glioblastoma cell proliferation and suppresses apoptosis [35]. It also plays a protective role in response to stress [36] and inflammation [37] and a recent study reported that up-regulation of miR-21 protected the kidney from the effects of delayed ischemic preconditioning (IPC) [38].

Considering the expression of miR-21 in plasma was less specific for AKI than in urine according to the results in this study, the high levels of plasma miR-21 in our patients may partly be derived from other organs other than the kidney. Indeed, previous studies have demonstrated high expression levels of MiR-21 in many organs and tissues after ischemic injury [39], [40]. It has also been demonstrated that microvesicles (MVs) derived from endothelial cells, platelets, and white blood cells, carry miRNAs and discharge them into circulation during and after infection and tissue injury, both of which may accompany cardiac surgery [41].

Conversely, urinary miRNA levels are thought to reflect kidney miRNA production, but relatively few reports have investigated this in the setting of AKI. Our estimates of the predictive power of miR-21 for the progression of AKI are comparable to those reported for other biomarkers. For example, a large multicenter study reported adjusted AUCs of 0.78, 0.79, 0.77, and 0.80, respectively for urine albumin to creatinine ratio, urine neutrophil gelatinase-associated lipocalin (NGAL), urine IL-18, and plasma NGAL, as markers of AKI progression [6]. The same clinical model forecast the risk of severe AKI as 0.75, which is equal to the performance of our clinical model.

One of the strengths of our study was the use of a control group of patients who did not develop AKI after cardiac surgery, instead of using healthy controls or comparator group without kidney disease. In addition we analyzed urine as well as plasma miR-21 levels. However, the present small single center study did not provide data on anuric/oliguric patients or those with a baseline eGFR <30 mL/min/1.73 m^2^. In addition, it was not possible to categorize miR-21 values into quintiles, which means our ORs may underestimate the association of different factors with the risk of AKI progression. It was not our purpose to investigate the molecular mechanisms underlying AKI progression and thus we were unable to distinguish whether the increased urinary miR-21 expression was derived from the renal tubules or was trans-renal.

In conclusion, this study provides evidence showing that levels of urinary and plasma miR-21 are associate with risk of severe AKI and other poor outcomes following cardiac surgery. Larger cohort studies are needed to confirm these findings, and experimental studies are required to identify the molecular pathophysiological mechanisms involved in the dysregulation of soluble miRNAs for AKI. More accurate identification and characterization of miR-21 with respect to AKI may result in marked progress in terms of therapeutic interventions.
